# P-1584. Diagnostic Stewardship of Cerebrospinal Fluid Testing: Impact on Quantity of Tests, Length of Stay, Antibiotic Prescriptions, and Cost

**DOI:** 10.1093/ofid/ofae631.1751

**Published:** 2025-01-29

**Authors:** Aaron Pathak, Sabra Shay, Todd M Lasco, Mayar Al Mohajer

**Affiliations:** Baylor College of Medicine, Houston, Texas; Premier, Charlotte, North Carolina; Baylor St. Luke's Medical Center, Houston, Texas; Baylor College of Medicine, Houston, Texas

## Abstract

**Background:**

While cerebrospinal fluid (CSF) cultures are the primary test of choice for meningitis, physicians can order a plethora of additional tests with varying levels of utility and cost. Some of these tests frequently have a high financial burden with little clinical impact. This study aims to assess whether limiting available CSF tests impacted the length of stay, empiric days of therapy (eDOT), and cost of testing in patients who received a lumbar puncture (LP) with subsequent infectious disease testing.

**Figure 1.**
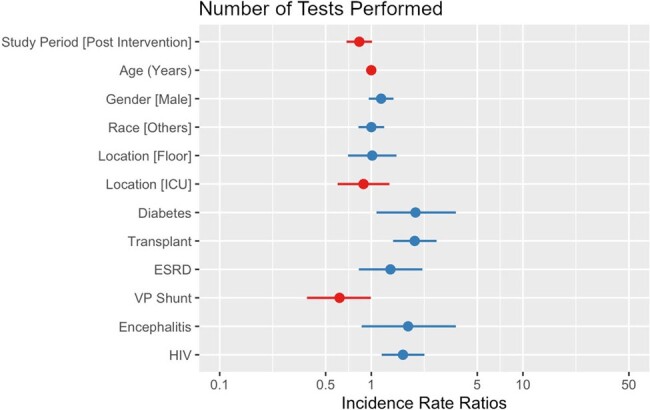
Number of tests performed (negative binomial regression)

**Methods:**

Our quasi-experimental study included patients from a quaternary academic medical center in Texas who received an LP with subsequent infectious disease testing. CSF testing was not restricted in the pre-intervention period (May 2023 - October 2023). In the post-intervention phase (December 2023 - February 2024), physicians were limited to ordering cell CSF count and differential, gram stain/bacterial culture, cryptococcal antigen, glucose, protein, meningitis/encephalitis panel, and VDRL if a serum RPR test was positive. Other CSF testing required consultation with the infectious disease team. Regression models were fitted to assess the impact of the intervention on study outcomes (negative binomial for the number of tests per patient, linear regression for the squared root of testing cost per patient and log length of stay, and zero-inflated negative binomial for eDOT).

**Figure 2.**
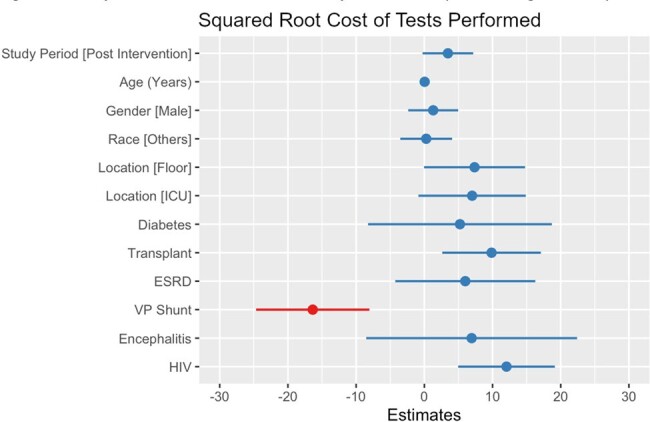
Squared root costs of tests performed (linear regression)

**Results:**

A total of 245 patients were included (158 pre-intervention and 85 post-intervention). After adjusting for confounders, the intervention did not significantly reduce the number of tests per patient (IRR= .83, p = .063, Figure 1), the squared root cost of tests per patient (B= 3.46, p = .068, Figure 2), eDOT (IRR= .55, p = .102, Figure 3) or the log length of stay (B= .14, p = .461, Figure 4).

**Figure 3.**
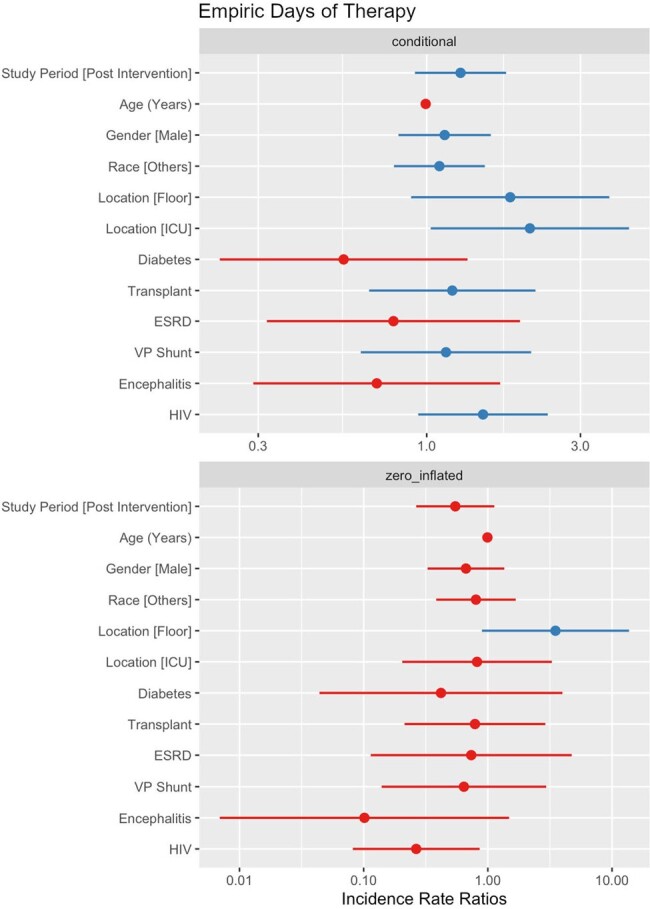
Empiric days of therapy (count and zero-inflated model)

**Conclusion:**

This intervention did not reduce the number of tests ordered or the cost of testing for patients with suspected meningitis. The decrease in unapproved tests was balanced by an increase in approved tests, with increased use of the meningitis/encephalitis panel preventing cost reduction. Future diagnostic stewardship interventions should limit expensive tests such as the meningitis/encephalitis panel to patients with a reasonably high pre-test probability.

**Figure 4.**
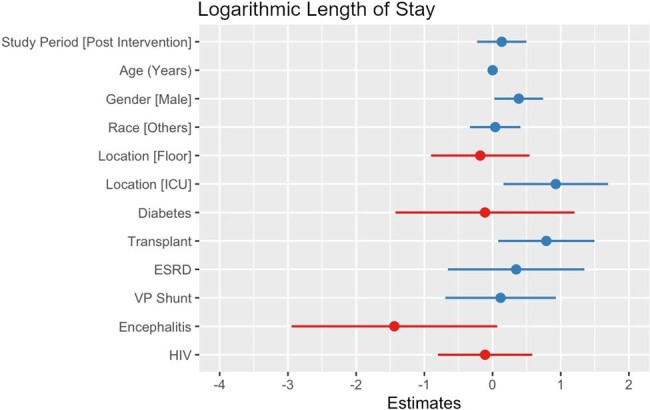
Logarithmic transformed length of stay (linear regression)

**Disclosures:**

**All Authors**: No reported disclosures

